# Ovine Toxoplasmosis Sero‐Status, Risk Factors Analysis, and Estimation of Haematological and Serum Biochemical Alterations

**DOI:** 10.1002/vms3.70220

**Published:** 2025-01-17

**Authors:** Shadan Hassan Abdullah, Hiewa Othman Dyary, Omer Ismael Dana, Mohamad Hasanvand

**Affiliations:** ^1^ Department of Microbiology College of Veterinary Medicine University of Sulaimani Sulaymaniyah Iraq; ^2^ Department of Basic Sciences College of Veterinary Medicine University of Sulaimani Sulaymaniyah Iraq; ^3^ Department of Surgery & Theriogenology College of Veterinary Medicine University of Sulaimani Sulaymaniyah Iraq; ^4^ Tehran University of Medical Sciences and Health Services Tehran Iran

**Keywords:** biochemical, haematological, sheep, risk factors, Sulaymaniyah, *Toxoplasma*

## Abstract

Toxoplasmosis is a significant food‐borne protozoal disease in humans and animals. The study aimed to find out *Toxoplasma* seropositivity in sheep, estimate epidemiological risk factors and assess haemato‐biochemical parameter changes. Blood samples were collected from 276 indigenous sheep in five districts surrounding Sulaymaniyah Province in the northern region of Iraq. Toxoplasmosis was determined by detecting anti‐*Toxoplasma* antibodies by ELISA. The impact of various risk factors on seropositivity was evaluated. Haematological and serum biochemical changes from naturally infected sheep were analysed using commercial kits. The overall seroprevalence of *Toxoplasma* antibodies among the studied population was 41.67%. Sharazoor district had a higher prevalence rate of 46.15% than Bakrajo, with the lowest frequency rate of 38.18%. Ewes with an abortion history had a higher risk for seropositivity of about 47.4% (OR = 1.4, 95% CI: 0.9–1.6). Sampling season and herd size are other risk factors, with higher seropositivity at 42.5% (OR = 1.1, 95% CI: 0.8–1.4) during the dry season and among small flocks ≤ 100 animals/farm at 43.6% (OR = 1.1, 95% CI: 0.7–1.4). *Toxoplasma* prevalence was correlated with animals' ages; a higher seropositivity of 43.3% was found among older sheep (OR = 0.8, 95% CI: 0.6–1.2). Seropositive sheep had significantly higher total leukocyte, lymphocyte and granulocyte counts but significantly lower haemoglobin, haematocrit and total erythrocyte counts. The serum levels of total protein and albumin were significantly reduced, with increased blood urea nitrogen and creatinine concentrations. Also, AST enzyme activity represents a higher value in positive sheep with toxoplasmosis. *Toxoplasma* infection among the ovine population is common. Its impact on livestock productivity should not be neglected. A potential risk for humans through infected sheep could be a hazardous source for public health.

## Graphical abstract

Toxoplasmosis is a significant protozoal disease that infects different hosts. A cross‐sectional study was performed to determine the serostatus of *Toxoplasma gondii* infection in association with haemato‐biochemical analysis and risk factor estimation. The study conducted from June to December 2023 involved 276 randomly selected indigenous sheep from five districts of Sulaymaniyah province, Iraq. The results demonstrate an occurrence rate of 41.67%. The estimated data represented an association of *Toxoplasma* infection with alteration in haemato‐biochemical values. The study also evaluated the impact of different epidemiological risk factors on the frequency rate of toxoplasmosis in sheep.

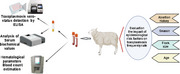

## Introduction

Toxoplasmosis is considered a prevalent food‐borne zoonosis of significance, involving both the veterinary field and public health (Opsteegh et al. 2016). It is caused by the apicomplexan parasite of the genus *Toxoplasma* (Djurković‐Djaković et al. 2019). *Toxoplasma gondii* is an obligate intracellular pathogen that can cause infection in almost all warm‐blooded animals, including humans (Castillo‐Cuenca et al. 2020).

*T. gondii* has three infective stages: tachyzoite, a rapidly dividing invasive stage; slowly dividing bradyzoites inside the tissue cysts; and the sporozoite environmental stage, protected inside an oocyst. *T. gondii* reproduces asexually in an intermediate host and sexually in a definitive host in cycles throughout its life cycle. *Toxoplasma* can spread by carnivorism between intermediate hosts and between definitive and intermediate hosts (Robert‐Gangneux & Dardé 2012).

Domestic cats and other cat species become definitive hosts; after ingestion of the infectious parasite stages, they might expel non‐sporulated oocysts, including bradyzoites, from the tissue of the intermediate hosts (Dubey 2010). Oocyst sporulation requires 1–5 days in the natural environment. Intermediate hosts contract the infection after consuming sporulated oocysts from plants, water or soil. Animal infections are thought to originate mostly from sporulated oocysts found in an environment contaminated by cat faeces and result in horizontal transmission (Čobádiová et al. 2013).

*Toxoplasma* is transmitted vertically during pregnancy and severely affects intermediate hosts, including sheep. Following the parasite's proliferation in the placenta and arrival at the foetus, the pathophysiology of abortion progresses. If abortion does not occur, it initiates irreversible congenital lesions (Dubey 1994).

In non‐pregnant ruminants, the infection usually shows no symptoms or for a few days may show unspecific symptoms including fever, appetite loss, nasal discharge, coughing and tachypnea for a few days and might remain unnoticed in traditionally rearing farms (Glor et al. 2013).

*T. gondii* is recognized as the main parasite associated with reproductive problems and abortion in small ruminants (Moreno et al. 2012). When primoinfection occurs during gestation, it might lead to foetal death, abortion, mummification, stillbirth or the birth of weak lambs, depending on the infection time. On the other hand, ewes with persistent infections typically do not pass on the parasite to their offspring, given that transplacental transfer and endogenous infection reactivation are rare in sheep (Dubey 2009).

The seroprevalence of *Toxoplasma* infection in domestic ruminants varies significantly across the globe, ranging from 3% to 92% in sheep (Tilahun et al. 2018). Various cross‐sectional serological studies are available on *T. gondii* in Iraq throughout the years; recent studies represented prevalence rates of 50.69% (Kareem et al. 2022), 53% (Shareefet al. 2022) and 18% (Bawi & Al‐Zubaidi 2024).

Sheep are susceptible intermediate hosts for *Toxoplasma*; the infection is spread by the consumption of contaminated food and water. Tissue cysts are developed in numerous organs, including the heart, liver, kidney, lungs, central nervous system and muscular tissue (Weiss & Kim 2014).

**Summary**

- The study represented that 41.67% of the examined sheep were pisitive for anti ‐*Toxoplasma* antibodies. FMD, while 54% of farmers avoided buying cattle from unidentified sources.
- Toxoplasmosis was reported from all selected farms belonging to the five regions of Sulaymaniyah province with various frequency rates.
- The study indicated the impact of various epidemiological risk factors on *Toxoplasma* seroprevalence rate.
- The study signified an association of toxoplasmosis with altration in several serum biochemical and hematological values.  addressing knowledge gaps and improving practices among farmers and wholesalers can help reduce the burden of FMD in this border region.

Toxoplasmosis in sheep causes economic loss through abortion among infected ewes, and the tissue containing cysts in meat becomes the source of human infection if taken undercooked (Weiss & Kim 2014). It seems that a simple and affordable method of lowering the risk of *T. gondii* transmission is to freeze meat for at least one night before eating by animals or humans (Dubey 2008).

Infection with *T. gondii* has harmful consequences that affect the animal's health status; it is connected with the disorder in vital organ functions, and the represented disturbances develop in the form of variations in haemato‐biochemical values (Mahboub et al. 2013). Estimation of haematology and serum biochemical profile can accurately assess an animal's health status (Ramprabhu et al. 2010). Biochemical parameters can assist in considering the host–parasite association (Al‐Kaysi et al.^2010^). Also, they are integral in representing the animal's natural physiological and nutritional status also and pathological disorders (Maina et al. 2015).

Various studies determined *Toxoplasma* seropositivity in small ruminants, while the estimation of its effects on haemato‐biochemical parameters was not well defined. So, this study attempted to clarify the impacts of various epidemiological risk factors on the Toxoplasma prevalence rate in sheep and explained the haemato‐biochemical changes.

## 1 and Methods

### 1.1 Study Area

The study was conducted in Sulaymaniyah Province from June to December 2023 in five selected districts. Sulaymaniyah is situated in the southwest of the Kurdistan region and north‐eastern part of Iraq; its 2800 ft above sea level. Its latitude is 35.566864 and the longitude is 45.416107. The climate characterized by a wide range of temperatures, including cold, rainy winters; neutral springs and autumns with moderate rainfalls; and hot, sunny and no rainfall in summer (https://www.climatestotravel.com/climate/Iraq).

### 1.2 Study Design and Sampling

For the study, 276 indigenous sheep from 20 different‐sized flocks with abortion history were selected for sampling; of these, 186 sheep were sampled during the dry and 90 during the wet season. They were divided into two age groups, ≤ 2 years old and > 2 years old.

Blood sampling was performed from the jugular vein of randomly selected animals. Approximately 7 mL of blood was drawn using a disposable sterile syringe. About 5 mL of blood was put into a tube without anticoagulant for serum biochemistry. The remaining blood was stored in a tube with EDTA for haematological analysis. A questionnaire was recorded to assess the effect of possible risk factors on seropositivity.

Samples were transported in an icebox to the research centre of the College of Veterinary Medicine, University of Sulaimani. The first group samples were centrifuged for 8 min at 3000 revolutions per minute. Sera aliquots were isolated and frozen at −20°C until they were analysed, and haematological analysis was performed for the second group.

### 1.3 Methodology

#### 1.3.1 Serological Analysis

For the detection of toxoplasmosis, all sera were verified for anti‐*T. gondii* IgG antibodies using a commercially available ELISA diagnostic kit from (Pishtazteb Company, Iran). The reagent preparation and the ELISA test were performed based on the manufacturer's recommendation. The result was read at 450 nm with an EL‐800 ELISA plate reader (Biotek Instruments Inc., USA), and absorbance appeared as optical density (OD).

#### 1.3.2 Blood Count and Haemato‐Biochemical Parameter Evaluation

Haematological analyses were performed using an automated haematology analyser (Medonic) to obtain the complete blood count values. The counted haematological parameters included total erythrocytes, haemoglobin concentration and haematocrit. Also, leukocytes, lymphocytes and granulocytes were measured (Lashari et al. 2020). The biochemical values included measuring the concentration of total proteins (TPs), albumin, urea, creatinine and aspartate aminotransferase (AST) enzyme in sera using Alpha Classic‐AT auto analyser.

### 1.4 Data Analysis

The data analysis was carried out by SPSS version 24.0, using Chi‐square (*χ* ^2^) of significance. The association between serological status and possible risk factors was done by calculating the odds ratio (OR) with 95% confidence intervals (CIs) based on the likelihood ratio. The calculated variables were also recorded as mean ± standard deviation.

## 3. Results

### 1 Sero‐Status of Toxoplasmosis in the Study Areas

The proportion of *T. gondii* seropositivity among the studied population was 41.67%, and animals with anti‐*T. gondii* IgG antibodies were found in all farms belonging to the study regions as viewed in Table 1. *Toxoplasma* seroprevalence ranged from 46.15% in the Sharazoor region to 38.18% in the Bakrajo region. With the seroprevalence of 43.33%, 40.00% and 39.34% in other regions of Tanjaro, Mawat and Bazian, respectively.

**TABLE 1:** Distribution of *Toxoplasma gondii* serostatus in sheep from Sulaymaniyah Province.

District	Total No.	No. of infected	Percentage of infected (%)	Confidence interval (95%)	Chi‐square value (*p* value)
Sharazoor	65	30	46.15	[21.10–24.89]	
Bazian	61	24	39.34	[20.88–25.12]	1.057
Tanjaro	60	26	43.33	[20.96–25.03]	0.90
Bakrajo	55	21	38. 18	[20.73–25.26]	
Mawat	35	14	40.00	[20.22–25.77]	
Total	276	115	41.67		

### 2 Risk Factors Analysis

The impact of various epidemiological risk factors on the Toxoplasma seroprevalence rate is illustrated in Table 2. According to the study data, abortion history, season and flock size are risk factors with an OR higher than 1. Also, higher seropositivity was found among aged sheep with no significant association.

**TABLE 2:** Ovine toxoplasmosis seroprevalence in association with epidemiological risk factors from Sulaymaniyah Province.

Determinants	Variables	Total No.	No. of infected	Percentage of infected (%)	*χ* ^2^ value [*p* value]	Odds ratio [95% CI]	Relative risk [95% CI]
Abortion history	Abortion	97	46	47.4	2.038	1.4	1.23
	No abortion	179	69	38.6	0.15	0.8–2.3	0.9–1.6
Season	Dry	186	79	42.5	0.152	1.1	1.06
	Wet	90	36	37.1	0.69	0.6–1.8	0.7–1.4
Flock size	Small (≤ 100 sheep/farm)	110	48	43.6	0.291	1.1	1.08
	Large (> 100 sheep/farm)	166	67	40.3	0.58	0.7–1.8	0.8–1.4
Age	≤ 2 years	105	41	39.0	0.478	0.8	0.90
	> 2 years	171	74	43.3	0.48	0.5–1.4	0.6–1.2
Total		276	115	41.67			

### 3 Estimation of the Haematological and Serum Biochemical Alteration

Analysis of haematological and serum biochemical changes in Table 3 exhibited a significant reduction in erythrocytes, haemoglobin and haematocrit value, while the total leukocytes, lymphocytes and granulocytes count were significantly higher in the infected sheep. The serum level of TP and albumin was significantly lower in the infected sheep with toxoplasmosis, with an increase in blood urea nitrogen (BUN), creatinine and AST levels.

**TABLE 3:** Analysis of haematological and serum biochemical parameters in *Toxoplasma* seropositive and negative sheep from Sulaymaniyah Province.

Parameter	Uninfected sheep	Infected sheep	*p* value
Erythrocytes (×10^6^/µL)	8.46 ± 0.07	7.52 ± 0.07	0.000
Haemoglobin (g/dL)	9.27 ± 0.08	7.89 ± 0.07	0.000
Haematocrit (%)	28.15 ± 0.26	23.69 ± 0.20	0.000
Leukocytes (×10^3^/µL)	9.00 ± 0.24	13.38 ± 0.28	0.000
Lymphocytes (×10^3^/µL)	4.51 ± 0.15	6.56 ± 0.22	0.000
Granulocytes (×10^3^/µL)	2.44 ± 0.14	3.41 ± 0.30	0.001
Total Proteins (g/dL)	7.10 ± 0.06	6.10 ± 0.05	0.000
Albumin (g/dL)	3.37 ± 0.03	2.58 ± 0.04	0.000
Aspartate transaminase (U/L)	82.99 ± 1.10	115.44 ± 2.95	0.000
Creatinine (mg/dL)	1.11 ± 0.01	1.72 ± 0.03	0.000
Urea (mg/dL)	25.27 ± 0.53	45.40 ± 0.43	0.000

## 2 Discussion

Toxoplasmosis is a parasitic infection in domestic and wild animals and has a zoonotic impact. Nearly one‐third of the world's population is infected with the parasite (Wang et al. 2021). Serological evidence of anti‐*Toxoplasma* antibodies in sheep has been found everywhere. In the current study, seroprevalence for *T. gondii* was 41.67%, and seropositive animals were found in all farms. The recent data indicated a decrease in the seroprevalence of toxoplasmosis from the previous estimate of 51.4% from Iraq's Sulaymaniyah Province by ELISA (Al‐Taie & Abdulla 2011). This decline could be due to variations in small ruminant populations, changes in climatic conditions, decreases in cat density or improvements in veterinary care in the study areas.

Studies from neighbouring countries represented various prevalence rate of 35.94% and 21% from Iran by Pashmforosh et al. (2023) and Firouzivand (2023), and in Turkey prevalence rate of 10% and 4.58% were found by Cak & Karatepe (2017) and Aktas & Aydin (2020), respectively.

Recent studies conducted in different parts of the world found that *Toxoplasma* prevalence rates among sheep to be as high as 66.3%, 61.96% and 50.64% from Switzerland, Mexico and Romania, by Basso et al. (2022), Alcalá‐Gómez et al. (2022) and Hotea et al. (2021), respectively. Moderate prevalence of 30%–50% for anti‐*T. gondii* antibodies in sheep were observed in other studies. A prevalence rate of 47% was reported from Poland (Moskwa et al. 2018) and 43.75% from Egypt (Khattab et al. 2022). The occurrence rate of toxoplasmosis in the current study is 41.67%, which was quite close to the previously reported data in Iran 41.5% (Chaechi Nosrati et al. 2020), 41.1% in Costa Rica (Villagra‐Blanco et al. 2019) and 42.7% from Brazil (Romanelli et al. 2021). However, indifferent to the study's findings, a lower prevalence of 35.6% was reported from Egypt (Fereig et al. 2022), 34.8% from Mongolia (Pagmadulam et al. 2020), 33.62% from Iran (Izadyar et al. 2019) and 33.7% from Ethiopia (Tilahun et al. 2018). Low seroprevalence rates of 10%–30% were detected from other locations. A prevalence of 25.6% was reported from Algeria (Abdallah et al. 2019), 27.4% (Lashari et al. 2020), 21.8% (Khattak et al. 2024) from Pakistan, and 17.8% from Mexico (Cruz‐Vázquez et al. 2020).

The serosurvey of toxoplasmosis represents variation between studied regions, which might be influenced by different epidemiological risk factors. Various factors can be attributed to the changes in *Toxoplasma* seroprevalence rates, including the livestock management system, density of feline hosts, environmental conditions, size of collected samples and stress factors (Selim et al. 2023).

Serological tests play a distinct role in investigating and diagnosing *T. gondii* by detecting IgG and IgM antibodies, which appear and are recorded about eight days postinfection. The IgM antibodies disappear entirely after a few months, while IgG antibodies can be present lifelong (Al‐Abodi 2019).

Environmental contamination might enhance seropositivity with *T. gondii*, mainly connected to food storage places and pastures with oocysts (Mahboub et al. 2013). The presence of cats is an essential factor in *Toxoplasma* infection on the farm. Uncontrolled cat movement in livestock shelters and pastures greatly increases the risk of oocyst spread and farm animal infection (Hotea et al. 2021).

Improper disposal of the placenta or aborted foetuses from *Toxoplasma*‐infected animals increases the opportunity for cats to be infected through consumption of these products, which makes the parasite life cycle continue with subsequent oocyst shedding (Hamilton et al. 2014). Due to the ability of an infected cat to shed a large number of oocysts, few cats are likely enough to quickly contaminate a big field area since an infected cat can discharge millions of oocysts (Tilahun et al. 2018).

Various risk factors, including age, breed, flock size, geographic location, climate rainfall and grazing area, might influence the *Toxoplasma* prevalence in sheep (Dubey et al. 2020). Age is considered as a factor making the animals more susceptible to *T. gondii* infection. According to the current study data, a higher seroprevalence rate of 43.3% (OR = 0.8, 95% CI = 0.6–1.2) was found in ewes over two years than in younger sheep, 39.0%, although the association was non‐significant. Other researchers also reported significantly higher seropositivity in aged ewes (Khattak et al. 2024; Khalife et al. 2022; Hotea et al. 2021; Kamal et al. 2019). Higher infection rates of toxoplasmosis in adults indicate horizontal transmission by ingesting sporulated oocysts from the environment (Rossi et al. 2011). That is why, with a progressive increase in age, animals have a greater chance of encountering *T. gondii* oocytes from contaminated food or water (Lashari et al. 2020).

Abortion is another studied risk factor, reported with a higher frequency rate of 47.4% among seropositive ewes (OR = 1.4, 95% CI = 0.9–1.6) than in non‐aborted 38.6%, which agrees with the previous reports by (Selim et al. 2023; Shehzad et al. 2022; Gebremedhin et al. 2014), as they found a correlation between *Toxoplasma* seropositivity and abortion history. Aborted herds showed higher positivity of 97.4% of anti‐*Toxoplasma* antibodies in lambs in a study by Movassaghi et al. (2016). In addition, PCR assay showed the existence of *Toxoplasma* in 64% of aborted foetuses' brain tissues (Shahbazi et al. 2019).

The present study also revealed an association between herd size and *Toxoplasma* seropositivity. A higher prevalence of 43.6% (OR = 1.4, 95% CI: 0.8–1.4) occurred in small farms compared to larger ones, 40.3%, which coincides with previous data by Gazzonis et al. (2016). Livestock production is widely dispersed in the study regions; semi‐extensive production is the primary farming system for small ruminants by winter housing until early spring and extensive pasture for the rest of the year.

Higher *T. gondii* seropositivity in the current study, 42.5%, was reported during the dry season (OR = 1.1, 95% CI = 0.7–1.4) compared to the wet season at 37.1%. A similar finding was reported by Gebremedhin et al. (2014), while the results of Khalife et al. (2022) disagreed when they reported a higher prevalence rate during the wet season.

Gradually decrease in temperature during the wet season, allowing the oocysts to maintain their viability and increase the chance of infection. It has been proposed that a decrease in the number of viable oocysts in the environment causes toxoplasmosis to decline during warmer and drier seasons (Logar et al. 2005).

Sheep sampled in the dry season were more likely to be seropositive than those sampled in the wet season. Variations in *Toxoplasma* transmission rate between seasons occur because more infections occur in the former wet months (where the climate is more suitable for the survival of the oocysts). In addition, IgG antibodies to *T. gondii* are long‐lasting in the host's bodies. The high seropositivity observed in the dry season might be due to the higher chance of infection during previous wet months (Gebremedhin et al. 2014).

The free‐ranging production systems in small ruminants make controlling the disease challenging. Nevertheless, education and training can readily avoid infection in the general community, which is crucial for immunocompromised people and pregnant women in particular (Gorji et al.^2018^). In addition, the number of cats that can shed oocysts should be kept to a minimum to decrease environmental contamination caused by oocysts (Dubey &  Jones 2008).

Haematological and serum biochemical values are crucial for estimating an animal's normal physiological status, management practice and nutritional and pathological conditions (Maina et al. 2015). The current study data revealed a significant effect of *T. gondii* on haematological parameters, given in Table 3. There was a significant reduction (*p* ≤ 0.05) in erythrocyte count, haemoglobin concentration and haematocrit in infected sheep. Other research also reported similar findings (Lashari et al. 2020; Shehzad et al. 2022).

In accordance with the study result, researchers also found low Hb values in infected sheep (Hanif & Tasawar 2016; Jawasreh et al. 2010). Other factors, including health status, stress, sex, age, co‐parasitic infection and environmental changes, might also be responsible for low Hb value in *T. gondii*‐infected sheep (Ramprabhu et al. 2010). Decreased erythrocyte count and low haemoglobin concentration are responsible for anaemia during *Toxoplasma* infection (Iewida & Cabanacan‐Salibay 2010).

Moreover, the haematological analysis of studied animals revealed a significant increase (*p* ≤ 0.005) of total leukocyte count, lymphocytes and granulocytes in infected sheep with toxoplasmosis. Similar findings were also reported by Shehzad et al. (2022) in camels. There was a significant increase in the level of serum AST in *Toxoplasma* seropositive sheep, which was consequent with the findings of Al‐Hussary & Al‐Zuhairy (2010) and Mahboub et al. (2013). Other studies also reported an increase in serum levels of ALT and AST enzymes from other hosts infected with *Toxoplasma*, including humans, camels and goats (Abdulameer 2020; Shehzad et al. 2022; Mahboub et al. 2013).

AST enzyme plays a significant role in amino acid metabolism. It is found in different body tissues, including erythrocytes, highly concentrated in the liver and heart muscles. Kidneys and skeletal muscles contain intermediate levels of AST, whereas other tissues have lower concentrations (Hanif &Tasawar 2016). The elevated AST level may be due to inflammatory conditions and necrosis in the host tissues, muscles, kidneys, liver and heart (Sivajothi et al. 2015). Alteration in levels of enzymes, such as ALT and AST, during toxoplasmosis indicates hepatic injury. In any occasion of hepatocellular dysfunction, the enzymes may leak into the circulation, increasing their value (Adeyemi & Akanji 2011).

The blood urea level was significantly higher among seropositive sheep with *Toxoplasma* infection than in seronegative animals. Such results were in line with other studies' previous reports (Shehzad et al. 2022; Mahboub et al. 2013). The detected increase in creatinine concentration from infected sheep was in harmony with observed data by Shehzad et al. (2022). In agreement with current data, a significant elevation in serum levels of urea and creatinine was reported in humans (Mahmood 2018).

Despite the adverse effect of *Toxoplasma* infection on liver function, toxoplasmosis can also be associated with increasing the level of BUN in infected sheep, which might be due to its harmful effects on the kidney, leading to the reduction in urea excretion from the body and subsequently increasing its level in serum (Dubey 1997). An elevation in BUN level indicates impairment in kidney function (Mahboub et al. 2013).

A reduction in serum levels of total protein and albumin in *Toxoplasma*‐infected sheep agreed with the finding of (Mahboub et al. 2013). However, an increase in serum TPs from seropositive ewes were reported in previous studies (Al‐Hussary& Al‐Zuhairy 2010; Castello et al. 2018). *Toxoplasma* infection results in a reduction in the serum levels of total protein and albumin, which is associated with the adverse effects of the parasites on the liver tissue that lead to extensive damage in the hepatocytes (Calderaro et al. 2009). Besides that, the metabolic changes cause a decrease in hepatic protein synthesis by reticuloendothelial cells in the liver (Bossi & Bricaire 2004). Furthermore, the harmful effect of *Toxoplasma* that results in kidney damage might lead to increased protein excretion in the urine and hypoalbuminemia (Stockham & Scott 2013).

*Toxoplasma* oocysts are highly resistant to common disinfectants, but temperatures above 60°C can kill them (Wainwright et al. 2007). Inadequate sanitation practices can contribute to the persistence of an infection and its horizontal transmission. The vertical pathway can also be involved in the spread and sustenance of infections (Gebremedhin et al. 2014). It is necessary to continuously monitor the *T. gondii* infection in animals to control the infection's spread and reduce the chance that humans could contract it by eating raw or undercooked meat (Khalife et al. 2022).

## 3 Conclusions

A high seropositivity of 41.67% among the sheep population in the study area indicates extensive exposure to *T. gondii*. The current findings highlighted the effects of various risk factors on *Toxoplasma* seroprevalence among sheep, and their identification facilitates reducing the impact of toxoplasmosis in ruminants. The parasitic nature of *T. gondii* that inhabits the blood and different tissues can result in an alteration in haemato‐biochemical values, which affects the host's general health status and is associated with reduced productivity, especially in sheep, through abortion and deaths of neonatal lambs. Applying epidemiological studies in other hosts is crucial to determining the state of *Toxoplasma* infection, especially in cats. Due to the impact of toxoplasmosis on public health status, essential efforts to reduce its influence on human health through the application of various strategies, including education plans, are important points to overcome such issues.

## Author Contributions

**Shadan Hassan Abdullah**: conceptualization, software, supervision, investigation, validation, project administration, funding acquisition, resources, visualization, formal analysis, writing–original draft, writing–review and editing. **Hiewa Othman Dyary**: data curation. **Omer Ismael Dana**: methodology. **Mohamad Hasanvand**: methodology. **Mohamad Hasanvand**: methodology, and **Othman Jamal Nasrulla**: sampling.

## Ethics Statement

This research study was reviewed and approved by the College of Veterinary Medicine Ethics Committee, University of Sulaimani, 2024. Informed verbal consent was obtained from animal owners before sampling.

## Conflicts of Interest

The authors declare no conflicts of interest.

### Peer Review

The peer review history for this article is available at https://publons.com/publon/10.1002/vms3.70220.

## Data Availability

The data that support the findings of this study are available on request from the corresponding author. The data are not publicly available due to privacy or ethical restrictions.

## References

[vms370220-bib-0001] Abdallah, M. C. , M. Kamel , B. Karima , et al. 2019. “Cross‐Sectional Survey on *Toxoplasma gondii* Infection in Cattle, Sheep, and Goats in Algeria: Seroprevalence and Risk Factors.” Veterinary Sciences 6: 63. 10.3390/vetsci6030063. 31295942 PMC6789635

[vms370220-bib-0002] Abdulameer, N. A. 2020. “The Effect of Toxoplasmosis on Some Blood Parameters of Gravid Women in Al‐Diwaniyah City.” International Journal of Pharmaceutical Research 12: 5–10. 10.31838/ijpr/2020.12.02.0042.

[vms370220-bib-0003] Abraham, E. G. , A. E. Moses , U. Motilewa , A. I. Uwah , E. I. Itina , and A. N. Umoh . 2021. “Ocular Toxoplasmosis Among Livestock Farmers and Raw Meat Handlers in Uyo, Nigeria.” Ethiopian Journal of Health Sciences 31, no. 2: 257–266. 10.4314/ejhs.v31i2.8. 34158777 PMC8188092

[vms370220-bib-0004] Adeyemi, O. , and M. Akanji . 2011. “Biochemical Changes in the Kidney and Liver of Rats Following Administration of Ethanolic Extract of Psidium Guajava Leaves.” Human & Experimental Toxicology 30: 1266–1274. 10.1177/0960327110388534. 21056949

[vms370220-bib-0005] Aktas, M. S. , and O. Aydin . 2020. “Seroprevalence and Associated Risk Factors of *Toxoplasma gondii* in Sheep in Erzurum Province, Eastern Anatolia Region.” Iranian Journal of Veterinary Research 21, no. 2: 126–129. 32849892 PMC7430368

[vms370220-bib-0006] Al‐Abodi, H. R. J. 2019. “Use of Immunological Methods to the Detection of Toxoplasmosis and Heat Shock Protein HSP70 in Men.” Journal of Parasitic Diseases 43: 234–239. 10.1007/s12639-018-01080-5. 31263328 PMC6570716

[vms370220-bib-0007] Alcalá‐Gómez, J. , L. Medina‐Esparza , I. Vitela‐Mendoza , C. Cruz‐Vázquez , T. Quezada‐Tristán , and J. Gómez‐Leyva . 2022. “Prevalence and Risk Factors of *Neospora caninum* and *Toxoplasma gondii* Infection in Breeding Ewes From Central Western Mexico.” Tropical Animal Health and Production 54: 225. 10.1007/s11250-022-03221-8. 35794282

[vms370220-bib-0008] Al‐Hussary, N. , and A. Al‐Zuhairy . 2010. “Effect of Toxoplasmosis and Brucellosis on Some Biochemical Parameters in Ewes.” Iraqi Journal of Veterinary Sciences 24, no. 2: 73–80. 10.33899/ijvs.2010.5609.

[vms370220-bib-0009] Al‐Kaysi, A. M. , R. A. A. Eid , and B. G. A. Fahmy . 2010. “Biochemical Studies on the Effect of Toxoplasma Infection on Liver and Kidney Functions in Mice.” Egyptain Journal of Comparative Pathology and Clinical Pathology 23, no. 1: 174–185.

[vms370220-bib-0010] Almeria, S. , and J. P. Dubey . 2020. “Foodborne Transmission of *Toxoplasma gondii* Infection in the Last Decade. An Overview.” Research in Veterinary Science 135: 371–385. 10.1016/j.rvsc.2020.10.019. 33148402

[vms370220-bib-0011] Al‐Taie, L. H. , and S. H. Abdulla . 2011. “Seroprevalance of Toxoplasmosis in Sheep and Goat: Iraq/Sulaimania.” Iraqi Journal of Veterinary Medicine 35: 16–24.

[vms370220-bib-0012] Basso, W. , F. Holenweger , G. Schares , et al. 2022. “ *Toxoplasma gondii* and *Neospora caninum* Infections in Sheep and Goats in Switzerland: Seroprevalence and Occurrence in Aborted Foetuses.” Food and Waterborne Parasitology 28: e00176. 10.1016/j.fawpar.2022.e00176. 36039091 PMC9418186

[vms370220-bib-0013] Bawi, M. K. , and I. A. H. Al‐Zubaidi . 2024. “Seroprevalence and Molecular Detection of *Toxoplasma gondii* in Sheep in Diyala Province, Iraq.” Assiut Veterinary Medical Journal 70, no. 183: 544–554. 10.21608/avmj.2024.303793.1304.

[vms370220-bib-0014] Bossi, P. , and F. Bricaire . 2004. “Severe Acute Disseminated Toxoplasmosis.” Lancet 364: 579. 10.1016/S0140-6736(04)16841-4. 15313352

[vms370220-bib-0015] Cak, D. D. , and B. Karatepe . 2017. “Seroprevalence of T*oxoplasma gondii* in Sheep From Nevşehir Province in Turkey.” Turkiye Parazitolojii Dergisi 41, no. 3: 148–151. 10.5152/tpd.2017.5245. 29035243

[vms370220-bib-0016] Calderaro, A. , S. Peruzzi , G. Piccolo , et al. 2009. “Laboratory Diagnosis of *Toxoplasma gondii* Infection.” International Journal of Medical Sciences 6: 135–136. 10.7150/ijms.6.135. 19319234 PMC2659488

[vms370220-bib-0017] Castello, A. , G. Bruschetta , R. P. Giunta , A. M. F. Marino , and A. M. Ferlazzo . 2018. “The Effect of *Toxoplasma gondii* on Plasma Serotonin Concentration in Sheep.” Veterinary World 11: 1500. 10.14202/vetworld.2018.1500-1505. 30532508 PMC6247880

[vms370220-bib-0018] Castillo‐Cuenca, J. C. , J. M. Díaz‐Cao , Á. Martínez‐Moreno , et al. 2020. “Seroepidemiology of *Toxoplasma gondii* in Extensively Raised Iberian Pigs in Spain.” Preventive Veterinary Medicine 175: 104854. 10.1016/j.prevetmed.2019.104854. 31790931

[vms370220-bib-0019] Chaechi Nosrati, M. R. , B. Shemshadi , P. Shayan , S. Ranjbar Bahadory , and A. Eslami . 2020. “Serological Determination of Toxoplasma gondii Among Sheep (Ovis aries) in Guilan Province, Iran.” Archives of Razi Institute 75, no. 4: 463–471. 10.22092/ari.2019.127291.1383. PMC8410149 33403841

[vms370220-bib-0020] Čobádiová, A. , K. Reiterová , M. Derdáková , et al. 2013. “ *Toxoplasma gondii*, *Neospora caninum* and Tick‐Transmitted Bacterium *Anaplasma phagocytophilum* Infections in One Selected Goat Farm in Slovakia.” Acta Parasitologica 58: 541–546. 10.2478/s11686-013-0171-5. 24338316

[vms370220-bib-0021] Cruz‐Vázquez, C. , I. De Velasco‐Reyes , and I. Vitela‐Mendoza . 2020. “Sero‐Prevalence of *Toxoplasma gondii* Infection and Associated Factors in Sheep From Jalisco, Mexico.” Journal of Parasitology 106: 392–394. 10.1645/19-176. 32556162

[vms370220-bib-0022] Djurković‐Djaković, O. , J. Dupouy‐Camet , J. Van der Giessen , and J. P. Dubey . 2019. “Toxoplasmosis: Overview From a One Health Perspective.” Food and Waterborne Parasitology 15: e00054. 10.1016/j.fawpar.2019.e00054. 32095624 PMC7034049

[vms370220-bib-0023] Dubey, J. P. 1994. “Toxoplasmosis.” American Veterinary Medical Association Journals 205, no. 11: 410–415. 7730132

[vms370220-bib-0024] Dubey, J. P. 1997. “Tissue Cyst Tropism in *Toxoplasma gondii*: A Comparison of Tissue Cyst Formation in Organs of Cats, and Rodents Fed Oocysts.” Parasitology 115: 15–20. 10.1017/s0031182097008949. 9226953

[vms370220-bib-0025] Dubey, J. P. 2008. “The History of *Toxoplasma gondii*—The First 100 Years.” Journal of Eukaryotic Microbiology 55: 467–475. 10.1111/j.1550-7408.2008.00345.x. 19120791

[vms370220-bib-0026] Dubey, J. P. 2009. “Toxoplasmosis in Sheep—The Last 20 Years.” Veterinary Parasitology 163: 1–14. 10.1016/j.vetpar.2009.02.026. 19395175

[vms370220-bib-0027] Dubey, J. P. 2016. Toxoplasmosis of Animals and Humans. Boca Raton: CRC Press.

[vms370220-bib-0028] Dubey, J. P. , and J. Jones . 2008. “ *Toxoplasma gondii* Infection in Humans and Animals in the United States.” International Journal for Parasitology 38: 1257–1278. 10.1016/j.ijpara.2008.03.007. 18508057

[vms370220-bib-0029] Dubey, J. P. , F. Murata , C. Cerqueira‐Cézar , O. Kwok , and C. Su . 2020. “Economic and Public Health Importance of *Toxoplasma gondii* Infections in Sheep: 2009–2020.” Veterinary Parasitology 286: 109–195. 10.1016/j.vetpar.2020.109195. 32979682

[vms370220-bib-0030] Fereig, R. M. , G. Wareth , H. H. Abdelbaky , et al. 2022. “Seroprevalence of Specific Antibodies to *Toxoplasma gondii*, *Neospora caninum*, and *Brucella* spp. in Sheep and Goats in Egypt.” Animals 12: 3327. 10.3390/ani12233327. 36496847 PMC9735859

[vms370220-bib-0031] Firouzivand, Y. 2023. “Prevalence of Toxoplasmosis in Sheep Slaughtered in Malekan Slaughterhouse by Modified Agglutination Test (MAT).” Journal of Zoonotic Diseases 7, no. 4: 393–397. 10.22034/jzd.2023.17025.

[vms370220-bib-0032] Gazzonis, A. L. , G. A. Garcia , S. A. Zanzani , L. M. O. Mora , A. Invernizzi , and M. T. Manfredi . 2016. “ *Neospora caninum* Infection in Sheep and Goats From North‐Eastern Italy and Associated Risk Factors.” Small Rumin Research 140: 7–12. 10.1016/j.smallrumres.2016.05.010.

[vms370220-bib-0033] Gebremedhin, E. Z. , M. Abdurahaman , T. Hadush , and T. S. Tessema . 2014. “Seroprevalence and Risk Factors of *Toxoplasma gondii* Infection in Sheep and Goats Slaughtered for Human Consumption in Central Ethiopia.” BMC Research Notes 7: 696. 25287190 10.1186/1756-0500-7-696 PMC4196015

[vms370220-bib-0034] Glor, S. B. , R. Edelhofer , F. Grimm , P. Deplazes , and W. Basso . 2013. “Evaluation of a Commercial ELISA Kit for Detection of Antibodies Against *Toxoplasma gondii* in Serum, Plasma and Meat Juice From Experimentally and Naturally Infected Sheep.” Parasites & Vectors 6: 85. 10.1186/1756-3305-6-85. 23561035 PMC3631134

[vms370220-bib-0035] Gorji, G. R. S. , M. Rassouli , and H. Staji . 2018. “Prevalence of Cerebral Toxoplasmosis Among Slaughtered Sheep in Semnan, Iran.” Annals of Parasitology 64, no. 1: 37–42. 10.17420/ap6401.130. 29716185

[vms370220-bib-0036] Hamilton, C. M. , F. Katzer , E. A. Innes , and P. J. Kelly . 2014. “Seroprevalence of *Toxoplasma gondii* in Small Ruminants From Four Caribbean Islands.” Parasites & Vectors 7: 449. 10.1186/1756-3305-7-449. 25249175 PMC4261777

[vms370220-bib-0037] Hanif, M. , and Z. Tasawar . 2016. “Hematological and Serum Biochemical Changes in Sheep Naturally Infected With *Toxoplasma gondii* in Southern Punjab (Pakistan).” Pakistan Journal of Life and Social Sciences 14, no. 1: 52–56.

[vms370220-bib-0038] Hotea, I. , V. Herman , E. Tîrziu , et al. 2021. “Seroprevalence and Risk Factors for *Toxoplasma gondii* Infection in Sheep and Goats From Romania.” Parasitologia 1: 36–44. 10.3390/parasitologia1020005.

[vms370220-bib-0039] Iewida, S. Y. P. , and C. Cabanacan‐Salibay . 2010. “Serologic Detection of *Toxoplasma gondii* Infection in Stray and Household Cats and Its Hematologic Evaluation.” Scientia Medica 20, no. 1: 76–82.

[vms370220-bib-0040] Izadyar, N. , B. Abd Nikfarjam , A. R. Esmaeili Rastaghi , S. A. Alizadeh , P. Heydarian , and M. Saraei . 2019. “A Serologic Study on *Toxoplasma gondii* Infection in Slaughtered Sheep and Goats in Qazvin Province, Iran.” Tropical Animal Health and Production 51: 1289–1293. 10.1007/s11250-019-01832-2. 30689155

[vms370220-bib-0041] Jawasreh, K. , F. Awawdeh , Z. B. Ismail , O. Al‐Rawashdeh , and A. Al‐Majali . 2010. “Normal Hematology and Selected Serum Biochemical Values in Different Genetic Lines of Awassi Ewes in Jordan.” Internet Journal of Veterinary Medicine 7, no. 2: 124–129.

[vms370220-bib-0042] Kamal, A. , J. U. Din , A. Kamil , et al. 2019. “Seroprevalence of *Toxoplasma gondii* in Sheep and Buffalo of District Charsadda, Khyber Pakhtunkhwa, Pakistan.” International Journal of Biosciences 14: 497–502. 10.12692/ijb/14.3.497-502.

[vms370220-bib-0043] Kareem, S. , N. J. Hadi , K. H. Shnawa , and B. A. Al‐Aboody . 2022. “Serological Detection of *Toxoplasma gondii* in Some Intermediate Hosts (Sheep and Goats) in Thi‐Qar Province, Southern Iraq.” Journal of Education for Pure Science 12, no. 2: 234–239. 10.32792/jeps.v12i2.205.

[vms370220-bib-0044] Khalife, S. , S. Moubayed , R. Mitri , R. Geitani , and D. El Safadi . 2022. “Seroprevalence and Risk Assessment of *Toxoplasma gondii* Infection in Sheep and Goats in North and Beqaa Governorates of Lebanon.” Veterinary World 15, no. 9: 2180–2185. 10.14202/vetworld.2022.2180-2185. 36341067 PMC9631372

[vms370220-bib-0045] Khattab, R. A. H. , S. M. Barghash , O. M. S. Mostafa , S. A. Allam , H. A. H. Taha , and A. A. E. B. Ashour . 2022. “Seroprevalence and Molecular Characterization of *Toxoplasma gondii* Infecting Ruminants in the North‐West of Egypt.” Acta Tropica 225: 106139. 10.1016/j.actatropica.2021.106139. 34562431

[vms370220-bib-0046] Khattak, I. , T. Usman , A. A. Swelum , et al. 2024. “ *Toxoplasma gondii* Infection in Small Ruminants From Khyber Pakhtunkhwa, Pakistan: Seroprevalence, Spatial Distribution and Associated Risk Factors.” Veterinary Parasitology: Regional Studies and Reports 47: 100979. 10.1016/j.vprsr.2023.100979. 38199687

[vms370220-bib-0047] Lashari, M. , U. Farooq , S. Mubeen , et al. 2020. “Seroprevalence of *Toxoplasma gondii* and Associated Hematological Alterations in Small Ruminants of DG Khan District of Southern Punjab, Pakistan.” Arquivo Brasileiro De Medicina Veterinária e Zootecnia 72: 1698–1704. 10.1590/1678-4162-11723.

[vms370220-bib-0048] Logar, J. , B. Šoba , T. Premru‐Sršen , and Ž. Novak‐Antolic̆ . 2005. “Seasonal Variations in Acute Toxoplasmosis in Pregnant Women in Slovenia.” Clinical Microbiology and Infection 11: 852–855. 10.1111/j.1469-0691.2005.01244.x. 16153265

[vms370220-bib-0049] Mahboub, H. D. , M. A. Helal , A. Eldaim Mabrouk , A. El‐Razek , and A. Elsify . 2013. “Seroprevalence of Abortion Causing Agents in Egyptian Sheep and Goat Breeds and Their Effects on the Animal's Performance.” Journal of Agricultural Science 5: 92. 10.5539/jas.v5n9p92.

[vms370220-bib-0050] Mahmood, O. I. 2018. “Effect of Toxoplasmosis on Hematological, Biochemical and Immunological Parameters in Pregnant Women in Tikrit City, Iraq.” Tikrit Journal of Pure Science 2, no. 21: 24–27.

[vms370220-bib-0051] Maina, S. M. , C. G. Gitao , and P. K. Gathumbi . 2015. “Hematological, Serological and Virological Findings in Sheep and Goats Experimentally Infected With Lineage III Peste Des Petits Ruminants Virus Isolates in Kenya.” Journal of Experimental Biology and Agricultural Sciences 13, no. 1: 81–88.

[vms370220-bib-0052] Moreno, B. , E. Collantes‐Fernández , A. Villa , A. Navarro , J. Regidor‐Cerrillo , and L. Ortega‐Mora . 2012. “Occurrence of *Neospora caninum* and *Toxoplasma gondii* Infections in Ovine and Caprine Abortions.” Veterinary Parasitology 187: 312–318. 10.1016/j.vetpar.2011.12.034. 22260901

[vms370220-bib-0053] Moskwa, B. , A. Kornacka , A. Cybulska , et al. 2018. “Seroprevalence of *Toxoplasma gondii* and *Neospora caninum* Infection in Sheep, Goats, and Fallow Deer Farmed on the Same Area.” Journal of Animal Science 96: 2468–2473. 10.1093/jas/sky122. 29659859 PMC6095263

[vms370220-bib-0054] Movassaghi, A. R. , M. Rassouli , A. Fazaeli , and M. R. Salimi‐Bejestani . 2016. “Outbreak of Ovine Congenital Toxoplasmosis in Iran, Confirmed by Different Diagnostic Methods.” Journal of Parasitic Diseases 40: 152–156. 10.1007/s12639-014-0467-x. 27065616 PMC4815860

[vms370220-bib-0055] Opsteegh, M. , G. Schares , R. Blaga , and J. van der Giessen . 2016. “Experimental Studies on Toxoplasma gondii in the Main Livestock Species (GP/EFSA/BIOHAZ/2013/01) Final Report.” EFSA Supporting Publications 13, no. 2: 995E.

[vms370220-bib-0056] Pagmadulam, B. , P. Myagmarsuren , N. Yokoyama , B. Battsetseg , and Y. Nishikawa . 2020. “Seroepidemiological Study of Toxoplama *gondii* in Small Ruminants (Sheep and Goat) in Different Provinces of Mongolia.” Parasitology International 74: 101996. 10.1016/j.parint.2019.101996. 31634631

[vms370220-bib-0057] Pashmforosh, M. , M. Sabaghan , M. Foroutan S. Haghi Karamallah , and A. Jamshidi . 2023. “The Seroprevalence and Risk Factors of *Toxoplasma gondii* Infection in Sheep in Kohgiluyeh and Boyer‐Ahmad Province, Southwest Iran.” Comprehensive Health and Biomedical Studies 2, no. 1: e146897.

[vms370220-bib-0058] Ramprabhu, R. , M. Chellapandian , S. Balachandran , and J. J. Rajeswar . 2010. “Influence of Age and Sex on Blood Parameters of Kanni Goats in Tamil Nadu.” Indian Journal of Small Ruminants 16: 249–251.

[vms370220-bib-0059] Robert‐Gangneux, F. , and M. L. Dardé . 2012. “Epidemiology of and Diagnostic Strategies for Toxoplasmosis.” Clinical Microbiology Reviews 25: 264–296. 10.1128/CMR.05013-11. 22491772 PMC3346298

[vms370220-bib-0060] Romanelli, P. R. , A. M. R. N. Matos , F. Pinto‐Ferreira , et al. 2021. “Anti‐*Toxoplasma gondii* and Anti‐*Neospora caninum* Antibodies in Sheep From Paraná State, South Brazil: Prevalence and Associated Factors.” Revista Brasileira de Parasitologia Veterinária 30, no. 1: e023220. 10.1590/S1984-29612021021. 33909837

[vms370220-bib-0061] Rossi, G. , D. Cabral , D. Ribeiro , et al. 2011. “Evaluation of *Toxoplasma gondii* and *Neospora caninum* Infections in Sheep From Uberlândia, Minas Gerais State, Brazil, by Different Serological Methods.” Veterinary Parasitology 175: 252–259. 10.1016/j.vetpar.2010.10.017. 21075529

[vms370220-bib-0062] Selim, A. , M. A. Marawan , A. Abdelhady , and M. H. Wakid . 2023. “Seroprevalence and Potential Risk Factors of *Toxoplasma gondii* in Dromedary Camels.” Agriculture 13: 129. 10.3390/agriculture13010129.

[vms370220-bib-0063] Shahbazi, G. , N. Hohooghi Rad , R. Madani , S. Matin , P. Mortazavi , and A. H. Jangjou . 2019. “ *Toxoplasma gondii* in Aborted Fetuses of Sheep in Ardebil Area, North‐West of Iran.” Iranian Journal of Parasitology 14, no. 3: 430–435. 31673261 PMC6815854

[vms370220-bib-0064] Shareef, S. O. , A. N. Yaseen , and A. H. Qader . 2022. “Seroprevalence of Toxoplasmosis in Sheep in the Northest of Baghdad Governorat.” Biochemical and Cellular Archives 22, no. 1: 747–752.

[vms370220-bib-0065] Shehzad, A. , A. Masud , T. Fatima , et al. 2022. “Seroprevalence of *Toxoplasma gondii* and Associated Alterations in Hematology and Serum Biochemistry of One‐Humped Camels (*Camelus dromedarius*) in Pakistan.” Veterinary World 15: 110. 10.14202/vetworld.2022.110-118. 35369577 PMC8924402

[vms370220-bib-0066] Sivajothi, S. , V. C. Rayulu , B. S. Reddy , and K. N. Kumari . 2015. “ *Trypanosomae evansi* Causes Thyroxin Imbalance With Biochemical Alterations in Wistar Rats.” Journal of Advanced Veterinary and Animal Research 2: 205–209. 10.5455/javar.2015.b68.

[vms370220-bib-0067] Stockham, S. L. , and M. A. Scott . 2013. Fundamentals of Veterinary Clinical Pathology. Hoboken, NJ: John Wiley & Sons.

[vms370220-bib-0068] Tilahun, B. , Y. H. Tolossa , G. Tilahun , H. Ashenafi , and S. Shimelis . 2018. “Seroprevalence and Risk Factors of *Toxoplasma gondii* Infection Among Domestic Ruminants in East Hararghe Zone of Oromia Region, Ethiopia.” Veterinary Medicine International 2018, no. 1: 4263470. 10.1155/2018/4263470. 29887984 PMC5985087

[vms370220-bib-0069] Villagra‐Blanco, R. , O. Barrantes‐Granados , D. Montero‐Caballero , J. J. Romero‐Zúñiga , and G. Dolz . 2019. “Seroprevalence of *Toxoplasma gondii* and *Neospora caninum* Infections and Associated Factors in Sheep From Costa Rica.” Parasite Epidemiology and Control 4: e00085. 10.1016/j.parepi.2019.e00085. 30666319 PMC6330264

[vms370220-bib-0070] Wainwright, K. E. , M. Lagunas‐Solar , M. A. Miller , et al. 2007. “Physical Inactivation of *Toxoplasma gondii* Oocysts in Water.” Applied and Environmental Microbiology 73: 5663–5666. 10.1128/AEM.00504-07. 17616618 PMC2042101

[vms370220-bib-0071] Wang, W. , Q. L. Gong , M. H. Li , et al. 2021. “The Prevalence of *Toxoplasma gondii* in Sheep in China: A Systematic Review and Meta‐Analysis.” Research in Veterinary Science 138: 19–29. 10.1016/j.rvsc.2021.05.016. 34090203

[vms370220-bib-0072] Weiss, L. M. , and K. Kim . 2014. Toxoplasma gondii: The Model of Apicomplexan‐Prospectives and Methods. UK: Elsevier.

